# Perspectives for a new drug candidate for Chagas disease therapy

**DOI:** 10.1590/0074-02760220004

**Published:** 2022-03-14

**Authors:** Maria de Nazaré Correia Soeiro

**Affiliations:** 1 Fundação Oswaldo Cruz-Fiocruz, Instituto Oswaldo Cruz, Laboratório de Biologia Celular, Rio de Janeiro, RJ, Brasil

**Keywords:** Trypanosoma cruzi, experimental chemotherapy, Chagas disease, drug candidates, proof of concept, phosphodiesterase inhibitors

## Abstract

Chagas disease (CD), a neglected tropical illness caused by the protozoan *Trypanosoma cruzi*, affects more than 6 million people mostly in poor areas of Latin America. CD has two phases: an acute, short phase mainly oligosymptomatic followed to the chronic phase, a long-lasting stage that may trigger cardiac and/or digestive disorders and death. Only two old drugs are available and both present low efficacy in the chronic stage, display side effects and are inactive against parasite strains naturally resistant to these nitroderivatives. These shortcomings justify the search for novel therapeutic options considering the target product profile for CD that will be presently reviewed besides briefly revisiting the data on phosphodiesterase inhibitors upon *T. cruzi*.

*State of the art* - First reported by Carlos Chagas in 1909, Chagas disease (CD), also named American trypanosomiasis, is a neglected tropical life-threatening illness caused by the protozoan *Trypanosoma cruzi.* CD represents a major cause of cardiac disease in the endemic areas, and it is estimated that more than 6 million people are infected mostly in poor areas of 21 countries of Latin America, including about 1 million women of reproductive age.^1^ It is estimated that over 75 million people are at risk of acquiring the disease, about 173,000 new cases occur annually with more than 75,000 deaths/year, however, less than 10% of infected people are diagnosed and less than 1% treated.^1^ ^,^ ^2^ In endemic areas, CD is mostly transmitted through vectorial route by the elimination of infective flagellated parasites into the feces/urine of blood-sucking insects known as ‘kissing bugs’ (triatomines, invertebrate hosts) after/during their bloodmeal*.* Parasites (metacyclic trypomastigotes) get access to vertebrate host cells when the individual scratches the area nearby the insect’s bite and/or through the direct contact with mucosal membranes.^3^ Thus, the trypomastigotes invade all nucleated cells by an endocytic process mediated by multiple ligand/receptor and signaling systems.^4^ Intracellularly, trypomastigotes escape from the parasitophorous vacuole, and convert to amastigote forms that are the multiplicative stage found in vertebrate hosts. After several binary division cycles, amastigotes differentiate back into trypomastigotes, which are the main forms released after the rupture of infected host cells.^4^ By blood and lymphatic flows, *T. cruzi* can parasitise different organs, and bloodstream forms can be ingested by invertebrate vectors during their bloodmeal. In the insect gut, trypomastigotes differentiate into epimastigotes, which are the replicative forms found in invertebrate hosts. After cycles of binary fission, epimastigotes convert to metacyclic trypomastigotes (metacyclogenesis), which are released into the insect feces and urine, completing the cycle.^4^ Other transmission routes include blood transfusion or organ transplants, mother-to-child (during pregnancy or childbirth), contaminated beverage, laboratory accidents, among others of minor relevance.^1^ ^,^ ^3^

In the last years, due to human migration, globalisation, urbanisation and broadening the insect vectors, CD has spread worldwide and now represents a global public health threat also in non-endemic regions such as North America, Australia, Asian and European countries.^1^ ^,^ ^2^ ^,^ ^3^ The disease has two sequential phases: acute and chronic phase. The acute phase is usually a self-limited febrile sickness (oligosymptomatic/ asymptomatic) with patent parasitaemia, lasting up to eight weeks. Due to a competent host immune response, the parasitic proliferation is controlled but not extinguished, and carriers evolve to a second step of the disease called chronic phase, which is long-lasting stage. As infected people may not display symptoms for many years, most is unaware of this silent and progressive chronic inflammatory disease that may last for the rest of the life, with parasites hidden in different organs.^3^ ^,^ ^5^ However, after years or even decades, by multiple cellular and molecular mechanisms not yet fully known but that involve several factors such as parasite persistence, altered balance of the host immune response and parasite and host genetic, around 30 to 40% of the infected individuals present cardiac and/or digestive disorders that can lead to death.^6^ ^,^ ^7^ *In vivo* imaging of experimental models demonstrated that *T. cruzi* infects all organs and tissues during the acute stage, whereas in the chronic stage, parasites are mainly confined to the colon, stomach, and skin.^8^ These authors reported a highly dynamic profile in infected mice with parasite foci appearing and disappearing in a short frame of few hours possibly due to infected host cells (such as phagocytes) being tracked from sites of persistence to peripheral tissues.^8^ A recent study reported a retrospective longitudinal study with 139 chagasic chronic patients which demonstrated that positive blood culture was associated with an increased risk of all-cause mortality and that *T. cruzi* persistence may impact CD pathogenesis and prognosis.^5^

Every year, CD kills more people in Latin America than any other parasitic disease, and despite of this sad scenario, its treatment still represents a big challenge. CD therapy is based on two old antiparasitic drugs that are in clinical use for at least half a century: nifurtimox (Nif)(5-nitrofurane, Bayer 2502; Bayer, Germany) and benznidazole (Bz) (2-nitroimidazole; Pharmaceutical Laboratory of the State of Pernambuco [LAFEPE], Brazil, ELEA/Mundo Sano, Argentina).^9^ Both present low efficacies especially in the later chronic stage, are contraindicated during pregnancy, cause severe side effects leading to high drop-out rates (treatment discontinuation ≈ 20%), and are inactive against parasite strains naturally resistant to both nitroderivatives.^9^ In fact, *T. cruzi* display high genetic diversity with multiple strains belonging to seven discrete type units (DTUs) and that result in different naturally resistance profile and distinct drug efficacy.^10^ ^,^ ^11^ Also, the recent pandemic Coronavirus disease 2019 (COVID-19) brought additional concerns adding a potential worsen prognostic for the chagasic carriers, impacting the most vulnerable populations who need public policies for health services. The pandemic interfered and reduced even more the access and use of the available existing therapy and diagnosis of *T. cruzi* infected patients.^12^

Regarding new treatment regimens of the approved drugs for CD, a prospective, multicentre, randomised trial - Benznidazole Evaluation for Interrupting Trypanosomiasis (BENEFIT) evaluated the efficacy and safety profile of Bz, comparing to placebo, in reducing clinical outcomes among patients with chronic Chagas’ cardiomyopathy.^13^ This clinical trial recruited 2854 individuals and showed that although significantly reduced the blood parasite detection, Bz fail to protect against cardiac clinical deterioration followed by a mean of 5.4 years.^13^ Some other clinical trials being conducted with Bz include the evaluation of new regimens with reduced doses and shorter periods of drug administration aiming to keep the drug activity while reducing its toxicity. In this sense, BENDITA, a phase II multicentre and randomised trial was conducted in Bolivia with chronic indeterminate chagasic carriers and evaluate the efficacy and safety of new Bz monotherapy regimens.^14^ The findings showed good efficacy and safety of Bz in shorter regimens (2-week treatment) with similar outcomes as compared to the current standard (8-week treatment).^14^ The findings supported the validation of a shortened Bz treatment regimen that could substantially improve tolerability and accessibility, but further studies are needed to confirm these results and longer follow up. The MULTIBENZ is a phase II, randomised, double-blind, and multicentre international clinical trial that recruited chronic chagasic carriers from four different countries (Argentina, Brazil, Colombia, and Spain) to receive Bz at different therapeutic schemes (150 mg/day for 60 days, 400 mg/day for 15 days, or 300 mg/day for 60 days - comparator arm). This trial aims clarify which is the most safe and efficacious Bz therapeutic regimen to sustain parasitic load suppression.^15^ Also, in the view that the level of parasitaemia is a risk factor for congenital transmission and a shorter and lower dose of Bz could reduce side effects and increase compliance, a clinical trial in Argentina (named Betty) is underway to test alternative therapy courses among seropositive women not previously treated and with a newborn during the postpartum period. This trial aims to compare the standard treatment (300 mg for 60 days) with the lower dose and shorter periods of drug administration (150 mg for 30 days), leading to a four-fold reduction in the total amount of the overall dose.^16^ Another current clinical trial is related to a nitroaromatic compound, fexinidazole, an oral drug recently registered to treat sleeping sickness. At 2014, a Phase II Proof of Concept study using fexinidazole was conducted in Bolivia but interrupted due to safety and tolerability issues. However, the obtained data suggested high efficacy rates of this nitroimidazole at the lowest dose tested and short treatment durations, and then, further investigation was started at four sites in Spain, and the results are expected to be available soon.^2^

As above discussed, there is an urgent need of a novel oral, safer, and efficacious drug for both stages of the disease (acute and chronic), with pediatric formulation and adapted to the field, as predicted in the Target Product profile (TPP) for CD.^2^ ^,^ ^17^ The current TPP lists the characteristics of a new drug that ideally include oral administration in acute and chronic patients (including pregnant women), for a short period of time, adapted to age (pediatric formulation), safer and more effective than the current options (Bz and Nif), effective against the different parasite DTUs and upon dorment/quiescent/persistent forms.^2^ ^,^ ^18^ In addition, new drugs must present desirable pharmacological properties (absorption, distribution, metabolism, excretion and transport - ADMET) that allow reaching plasma and target organ levels in therapeutic concentrations.^17^

To improve the chances of success, a new drug candidate must follow a pipeline with a set of well-defined progression criteria (Target Candidate Profile or TCP) assessed by well standardised and validated models through screening cascades. This cascade must take into consideration the variations of efficacy due to differences in *T. cruzi* strains drug response and replication rates, the mode of action (active or not upon low metabolic forms and persister parasites) as well as desirable drug metabolism and pharmacological characteristics (optimal pharmacokinetic/pharmacodynamic profile).^18^ Aiming to contribute for the development of more robust and standardised protocols of anti-*T. cruzi* drug screening, in 2010, a technical note reported in detail a consensus drug pipeline derived from a two-days brainstorm meeting organised by Fiocruz and DND*i*, joining different experts from diverse knowledge areas of CD.^19^ After more than one decade, this pipeline has been updated by gathering new knowledges (from clinical and pre-clinical data). Usually, the pipeline starts with the identification, by primary screening of active compounds at a fixed concentration, of hits (e.g., EC_50_ < 5 µM, reaching max activity > 95%, selectivity > 10). These hits are next optimised and submitted to additional assays by multi-parametric analysis and after further rounds of optimisation, lead compounds may be generated (e.g., EC_50_ < 1 µM, reaching max activity > 95%, selectivity > 50, with metabolic stability *in vitro*, adequate lipophilicity and solubility, free C_min_ above EC_50_/EC_90_ for at least 8 hours, and with no adverse effect plus significant reduction of parasitaemia, at 50 mg/kg by oral dosing for 5 days in *in vivo* mouse experimental models).^18^ These lead compounds might progress as preclinical (e.g., 100% control of parasite burden after immunosuppression of acute and chronic mouse models of CD without major safety issues and reaching a human dose prediction > 10 mg/kg/day) and then as clinical drug candidates.^18^

Also, whenever possible, during *in vivo* pre-clinical studies, blood samples may be collected during the life-experiment to evaluate PK/PD in infected animals.^17^

However, despite the large amount of pre-clinical studies screening different classes of natural products and synthetic compounds, only few drugs moved to clinical trials, which included repurposing studies with two anti-fungi triazoles inhibitors of the sterol 14a-demethylase (CYP51): Posaconazole (POSA) and E1224, the later a prodrug of fosravuconazole, an antifungal discovered by Eisai Ltd (Japan).^9^ Although showing very promising in *in vitro* and *in vivo* assays, both CYP51 inhibitors fail to sustain efficacy in clinical trials.^13^ ^,^ ^14^ ^,^ ^18^ The discrepancy of pre-clinical and clinical outcomes of both azoles raised several concerns regarding the lack of translation of pre-clinical (*in vitro* and *in vivo*) and clinical trials. One explanation proposed could rely on the mechanism of action of these triazoles. The activity of ergosterol biosynthesis inhibitors is time-dependent and depletion of parasite ergosterol pools are essential to kill *T. cruzi* that, similarly as fungi, depends on endogenous synthetised sterols, essential components of plasma membranes and precursors of regulatory molecules acting in cellular growth, differentiation, and development.^20^ Fast sterol production is critical for rapidly multiplying cells such as amastigotes of *T. cruzi* and thus CYP51 inhibitors are usually very effective against intracellular forms (at nanomolar range) and much less active upon parasite forms with reduced metabolisms such as trypomastigotes and quiescent parasites that sustain the parasitism when the therapy is finished.^9^

Other argument that could explain the lack of pre-clinical and clinical outcomes for POSA and E1224 is the higher drug exposure attained in animal models as compared to humans since pharmacokinetic studies revealed that drug exposure was 5-10-fold lower in chagasic patients than in mice.^21^ Thus, it has been claimed that doses and time of treatment of chronic chagasic carriers could have been insufficient to reach the desirable plasmatic and tissular drug concentrations required to kill the parasites.^21^

On the other hand, the repurposing of antifungal azoles for treatment of protozoan infections may not be the best choice due to the low sequence identity (≈ 25%) of trypanosomatid and fungal CYP51s, and thus more specific irreversible inhibitors are attractive.^22^ In this sense, novel imidazole-based scaffolds of protozoan CYP51 inhibitors have been assayed and demonstrated potent anti-parasitic effect.^23^ VNI ((R)-N-(1-(2,4-dichlorophenyl)-2-(1H-imidazol-1-yl)ethyl)-4-(5-phenyl-1,3,4-oxadiazol-2 yl)benzamide and its derivative, VFV [(R)-N-(1-(3,4′-difluorobiphenyl-4-yl)-2-(1H-imidazol-1-yl)ethyl)-4-(5-phenyl-1,3,4-oxadiazol-2-yl)benzamide] - designed to fill the deepest portion of the CYP51 substrate binding cavity - were proven active upon mouse experimental model of *T. cruzi* acute infection (Y strain).^24^ VFV was more potent than VNI in both male and female infected animals, causing a > 99.7% peak parasitaemia reduction, but both resulted in 100% animal survival.^24^ In another set of studies, mice infected with Colombiana strain (a highly naturally resistant *T. cruzi* strain) and treated orally with VFV alone for 60 days presented parasitaemia suppression and 100% of animal survival but parasite relapse after interruption of drug administration.^25^ Importantly, co-administration of VFV plus Bz (given simultaneously) led to parasitological cure in 70% of the animals infected by Colombiana strain, claiming in favor of the promising aspect of the drug co-administration in improving the efficacy of therapeutic arsenal against *T. cruzi*.^25^

The lack of translation among the pre-clinical and clinical outcomes of POSA and E1224 also raised discussions related to the need of more reproducible experimental models, readouts, design of novel anti-*T. cruzi* screening cascades and more accurate protocols that could minimise these translation gaps.^17^ ^,^ ^18^ Some of these drug screening protocols investigate sterile cure including *in vitro* assays of sterile cidality^26^ besides *in vivo* bioluminescence imaging system to detect parasite recrudescence in drug-treated and untreated mouse experimental models of acute and chronic CD, under immunosuppression regimens.^27^ These assays reported the limited efficacy of POSA (and other similar CYP52 inhibitors) to cure *T. cruzi* experimental infections.^26^ ^,^ ^27^

Wash-out assays investigate *in vitro* sterility by detecting parasite relapse after compound withdraw. Under this approach, it was reported that even after extensive periods of incubation, POSA was unable to extinguish the parasitism of *T. cruzi*-infected cultures even using rapidly replicating parasite strains.^28^ Similar results were found using nucleoside derivatives assayed against *T. cruzi* infection.^29^ ^,^ ^30^ As kinetoplastids parasites are incapable of *de novo* purine synthesis, depending on purine salvage pathways to acquire and process these elements from the hosts, purine nucleoside analogues represent an important source of novel antiparasitic agents. One of these promising compounds (7-aryl-7-deazapurine 3′-deoxyribonucleoside derivative) gave parasitaemia suppression and 100% animal survival in acute mouse models of experimental CD but even after long periods of drug exposure, failed to induce cure *in vivo* identified after cycles of cyclophosphamide administration. In fact, *in vitro* wash-out experiments showed that that although parasite release into the supernatant of infected cultures was substantially lessened (> 94%), parasite recrudescence was achieved after compound withdraw.^30^ The bulk of these results (cure failure *in vitro* and *in vivo*) strongly suggest the occurrence of a heterogeneous parasite population *in vitro* that is not affected by the drug exposure, possibly quiescent/dorment/persister-like stages.^28^ ^,^ ^29^ ^,^ ^30^

Persister-like cells may tolerate high drug pressure for long periods of exposure, being able to resume growth after drug withdrawal, leading to treatment failures without selection of genetically heritable mutations.^28^ This biological phenomenon already reported in different microbes and tumor cells does not seem to be related to drug resistance but rather to drug tolerance.^31^ *In vitro* and *in vivo* data demonstrated evidence of non-replicating intracellular amastigotes of *T. cruzi* present in host cells alongside actively dividing amastigotes. These non-replicating and replicating amastigotes may differentiate into trypomastigotes in the same host cell and derived trypomastigotes which may be able to invade new host cells and re-establish both actively dividing and non-dividing progenies.^31^ Recent studies reported amastigotes of *T. cruzi* resuming proliferation after removal of Bz without changes in drug susceptibility *in vitro* as compared to parental parasite population.^32^ Dormant/quiescent/persisters amastigotes may evade host immune surveillance, acting as reservoirs in different tissues and organs and their occasional reactivation could at least in part, explain the dynamic nature of *T. cruzi* infection with parasite colonisation and location in different times of infection in distinct host organs.^31^ In this sense, it has been suggested that new trypanocidal drugs could be more efficacious under longer periods of administration, possibly using an intermittent scheme of therapy. The use of Bz under an intermittent modified treatment regimen at higher doses (2.5-5-fold the standard daily dose) given once per week eliminated actively replicating parasites as well as the residual, transiently dormant parasites, extinguishing *T. cruzi* infection in mouse models of acute and chronic experimental CD.^33^

Also, other important topic is to assess different *T. cruzi* strains and DTUs to identify active compounds upon these diverse parasite populations and that could be used in all geographic regions and endemic areas as above described as preconised for a new CD drug.^2^ In this sense, recent data using a panel of different *T. cruzi* strains representatives of the major parasite DTUs, demonstrated that PAH179 strain (belonging to DTU V) was less susceptible to POSA *in vitro* as compared to other parasite strains. The lack of sensitivity of PAH179 to POSA was attributed to its slow doubling time, which reinforces the idea that the antiparasitic effect of this CYP51 inhibitor arises after multiple rounds of parasite division, also corroborating the previous findings regarding its low potency towards non-replicative forms like the trypomastigote stage.^28^ The inability to kill trypomastigotes of *T. cruzi* was also correlated to the parasitaemia recrudescence and lack of sterile cure of some nucleoside analogues.^29^ The bulk of these data demonstrate the relevance of targeting both the intracellular multiplicative amastigotes and the non-replicative trypomastigote forms, the two relevant forms for mammalians infection by *T. cruzi* aiming to identify promising drug candidates for CD with curative effect *in vitro* and *in vivo.*

Besides assessing different *T. cruzi* strains and DTUs, an attention must be given to the source of the host cell and the metabolic and environmental heterogeneity as they can impact in the screening of novel drug candidates for CD. A systematic study of host cells interference with *T. cruzi* susceptibility using a small library of distinct compounds has been conducted using different cell lines as host cells. The findings demonstrated that the rates of infection as well as the susceptibility to the studied compounds (including Bz) varied greatly depending on the host cell and the parasite strain.^34^ While revisiting pyrazolo[3,4-d]pyrimidine nucleosides as anti-*T. cruzi* agents it has been found that a derivative (named cp 44) highly active against intracellular forms present in primary cultures of cardiac cells and mammalian cell lineages had its anti-parasitic effect vanished when primary mouse macrophages were used as host cells, likely due to permeability issues.^29^ The different levels of drug permeability into different cells and organs could also explain why some compounds (such as this nucleoside derivative, cp44) do not cure *T. cruzi* infection experimentally.^29^ ^,^ ^30^ Also, a n-phenyl-substituted analogue (DB569) of furamidine (DB75) presented different outcomes *in vitro* depending on the nature of the host cell employed for *T. cruzi* infection.^35^ DB569 has equivalent DNA binding properties compared to DB75 but is more potent upon *T. cruzi* (EC_50_ levels in low-micromolar range). The substitution of the amidine by the phenyl group enhanced the lipophilicity of the compound and the modulation of these physicochemical parameters could have improved its delivery to the intracellular parasites, explaining, at least in part, the improved anti-parasitic activity.^35^ While comparing the trypanocidal activity upon intracellular amastigotes (Dm28c clone) present in mouse cardiac cell cultures and peritoneal macrophages, a greater antiparasitic effect was found (at 2 µM) upon the parasites within professional phagocytes (> 75% parasitism reduction) as compared to cardiac cultures (≈ 26%),^35^ once more corroborating the idea that drug activity varies according to parasite strain, host cell type, and metabolic conditions and environment.^34^

Still regarding the mammalian host cells, phenotypic assays using three-dimensional cultures (3-D) have been used in several drug discovering programs as likely resemble *in vivo* biological behavior than 2D matrices, reproducing more closely cellular microenvironments.^36^ Recently, cardiac spheroids from primary cultures of mouse heart were used to evaluate host cell cytotoxicity, anti-*T. cruzi* activity and inflammatory profile after Bz treatment *in vitro*.^36^ The data demonstrated that Bz control parasite infection in cardiac organoids and modulate the release of inflammatory mediators produced by the own heart environment such as TNF and IL-6, relevant for *T. cruzi* infection.^36^ The overall findings contribute to the better knowledge to define more precise and accurate *in vitro* phenotypic assays to be used in pipelines for new drug candidates for CD therapy.

*Phosphodiesterases as target for novel anti-T. cruzi agent* - As above described for the CYP51 azole inhibitors, the identification of new therapeutic options also implies the analysis of drugs already licensed for other illnesses (“repositioning”) and that share common mechanisms of action and cellular targets. Such approach makes possible to reduce the time and costs of safety studies (pre-clinical and clinical) and has been greatly encouraged in the search for alternative therapies for NTDs.^9^ As also above reported, repurposing may be associated with another promising strategy; the combination and/or co-administration of drug candidates and reference drugs and even hybrid molecules, aiming to promote efficacy, reduce adverse effects and costs.^25^ In this sense, a 4-year, EU-funded project, the consortium PDE4NPD (https://cordis.europa.eu/project/id/602666/reporting) focused on new therapies for CD, sleeping sickness, leishmaniasis, and schistosomiasis by targeting parasite phosphodiesterases (PDEs). PDEs are highly conserved hydrolases that regulate the intracellular levels of cyclic nucleotides (cAMP and cGMP) by catalysing the hydrolysis of these second messengers, to their inactive 5’-AMP and 5’-GMP forms.^37^ These second messengers play a role in several cellular functions such as signal transduction and gene expression, cell proliferation and differentiation, inflammation, and cell death, among others.^38^ The signaling cascades promoted by cAMP is well conserved, being present from human to bacteria, including Kinetoplastida parasites that display four families of PDEs class I (PDEA, PDEB1/B2, PDEC, and PDED) that have been implicated in cell division, osmoregulation and virulence.^39^ ^,^ ^40^ The high level of conservation between PDEs from mammalians and trypanosomatids has the advantage as PDEs are already pharmacological entities in clinical use and some of the homologues seem to be essential in trypanosomatids.^39^ ^,^ ^40^ Trypanosoma PDEs were pharmacologically validated as therapeutic target for *T. brucei* using the tetrahydrophthalazinone NPD-001 (previously known as Cpd A). NPD-001 triggered a dose-dependent increase in the cAMP intracellular content in bloodstream forms, leading to immediate inhibition of proliferation and loss of viability after 72 h of incubation.^41^ Our group recently reported the anti-*T. cruzi* effect of 12 new phthalazinone PDE inhibitors against different parasite strains and forms (intracellular and bloodstream forms) besides also reporting, for the first time, the expression of the five TcrPDEs in all parasite stages.^42^ In this study, four out of 12 compounds were effective against intracellular forms of Tulahuen and Y strains (EC_50_ ≤ 10 µM) and three were more or displayed similarly activity as Bz against bloodstream forms (Y strain). *In vitro* combination with one of the most active phthalazinone (NPD-040) plus Bz on both parasite forms demonstrated a borderline synergistic profile (ΣFIC = 0.58) against intracellular amastigotes but no interaction (ΣFIC = 1.27) upon bloodstream forms. NPD-040 disrupted Golgi apparatus, induced a swollen flagellar pocket and caused an autophagic phenotype in treated bloodstream trypomastigotes after a short period of compound incubation (2 h).^42^ Both NPD-001 and NPD-040 raised the intracellular cAMP levels in both parasite forms, which was also released into the extracellular milieu.^42^ Another phosphodiesterase inhibitor, the tetrahydrophthalazinone named NPD-008 was investigated upon *T. cruzi in vitro*.^43^ NPD-008 was active against different forms and strains of *T. cruzi* (EC_50_: 6.6 to 39.5 mM) and triggered cAMP levels in treated parasites. Against bloodstream trypomastigotes, NPD-008 reached an EC_50_ of 6.6 ± 1.1 μM after 24 h incubation, being twice as active than Bz (EC_50_, 12.9 ± 1.9 μM).^43^ The combo NPD-008 plus Bz gave a synergistic and no interaction profile for bloodstream and amastigotes with ΣFICI values of 0.51 and 1.19, respectively, as already noticed for other phthalazinone PDE inhibitor such as NPD-040.^42^ These data are very promising since combined therapy is a valuable tool in improving treatment efficacy while reducing dose levels and toxicity and preventing the development of drug resistance.

Another class of PDE inhibitor evaluated was the heterocyclic pyrazonoles. A recent *in vitro* phenotypic screening identified a 4-bromophenyl-dihydropyrazole dimer as an anti-*T. cruzi* hit and 17 novel pyrazolone analogues with variations on the phenyl ring were investigated. A greater anti-parasitic effect was observed against intracellular amastigotes (EC_50_ values ranging from 0.17 to 3.3 µM on Tulahuen and Y strains) as compared to bloodstream trypomastigotes (Y strain).^44^ Previous validation studies performed by the measurement of cAMP levels showed that untreated amastigotes have higher ability to efflux this second messenger than untreated bloodstream forms, which may influence, at least in part, the different susceptibility profile of trypomastigotes and amastigotes, as observed with the exposure of some PDE inhibitors.^42^ ^,^ ^44^ The pyrazoles NPD-227 altered the morphology of bloodstream trypomastigotes inducing a rounding effect that impaired their invasion into the cardiac host cells, denoting that although not able to induce a rapid lysis, this pyrazole reduced the parasite infective fitness.^44^ The rounding effect induced by NPD-227 in bloodstream forms may be related to an osmo-deregulation, an effect already reported during PDEC inhibition in *T. cruzi* ^45^ As noticed with other classes of PDE inhibitors such as phthalazinones,^42^ pyrazolones caused profounds insults in Golgi, flagellar pocket, and plasma membrane of *T. cruzi* likewise suggestive of osmotic stress attributed to increased cAMP levels that was observed in treated parasites, validating the targeting of at least one PDE parasite isoforms.^45^ Due to the high potency and selectivity *in vitro* and its additive interaction with Bz, the pyrazolone NPD-227 was further investigated in experimental mouse model of *T. cruzi* (Y strain) of acute infection. Animals received oral administration for 5 days using 10 mg/kg NPD-227 plus Bz at 10 mg/kg, starting the therapy at the parasitaemia onset. The combo not only reduced parasitaemia (> 87%) but also protected against mortality (> 83% animal survival) corroborating the superiority of combos as compared to monotherapy schemes and supporting the hypothesis that pyrazolones display promising anti-*T. cruzi* effect, especially when used in combo with Bz.^45^

Finally, imidazoles designed as inhibitors of PDEs were identified in screenings against trypanosomatids that prompted a hit optimisation and structure-activity relationship to improve some drug-like properties such as aqueous solubility through increased polarity.^46^ Then, a series of 26 new imidazoles, closely related to the original hits but with fewer aromatic rings and containing a urea moiety, were synthesised, and assessed against *T. cruzi*. Our results revealed additional hits with EC_50_ values in the same range of the reference drug (Bz) and one representative hit, named compound 9 (EC_50_ = 1.2 µM) was about ten-fold more active against bloodstream trypomastigotes than Bz (EC_50_ = 12.9 µM).^46^ Ultrastructural analysis of drug-treated bloodstream trypomastigotes showed cellular changes consistent with autophagy and osmotic stress, mechanisms already previously linked to cAMP signaling as also observed for other PDE inhibitors.^42^ ^,^ ^43^ ^,^ ^44^ ^,^ ^45^ ^,^ ^46^ Compound 9 raised both cellular and supernatant cAMP levels, confirming inhibition of *T. cruzi* PDE(s).

The bulk of our results support the further investigation of PDE inhibitors as anti-*T. cruzi* agents as it represents a successful repurposing of research into inhibitors of mammalian PDEs.^42^ ^,^ ^43^ ^,^ ^44^ ^,^ ^45^ ^,^ ^46^

*In Conclusion* - Chagas disease poses as a global public health threat and the available therapies, introduced more than half century in clinical use, are far from being acceptable, mostly due to their undesirable effects and lack of efficacy in the later chronic phase of the disease. In this sense, new drugs and/or alternative therapies are urgently required including the possible use of reproposing strategies and combined therapy that allow the identification of novel drug candidates safer, effective, to be developed under a shorter period and with lower costs, also avoiding the potential of triggering drug resistance. In this context, during pre-clinical studies, it is largely desirable perform accurate protocols and use robust experimental models aiming to identify hit and lead compounds aligned to the current TPP for Chagas disease and that reduce the failure of translation among preclinical studies and clinical trials. In this context, some well-validated phosphodiesterase inhibitors present promising activity against *in vitro* and *in vivo* infection by *T. cruzi*, especially when combined with the reference drug for CD, benznidazole. As (i) the control of cyclic nucleotide metabolism is critical in kinetoplastids, and (ii) the catalytic domains of their PDEs align closely with human homologues qualifying their potential repurposing as potential antiparasitic agents, the repurposing of PDE inhibitors as anti-*T. cruzi* agent represents an interesting strategy successfully used for a large spectrum of clinical conditions including intermittent claudication, chronic obstructive pulmonary disease, erectile dysfunction, among others.^47^
